# Cholestatic Hepatitis in Young Migrants Exposed to Fuel Vapours: An Emerging Toxicity From the Mediterranean Sea

**DOI:** 10.1111/apa.70550

**Published:** 2026-04-17

**Authors:** Antonio Corsello, Simone Maria Calogero Gramaglia, Giuseppe Gramaglia, Gerlando Verruso, Giusy Ranucci

**Affiliations:** ^1^ Department of Clinical‐Surgical, Diagnostic and Pediatric Sciences University of Pavia Pavia Italy; ^2^ Department of Pediatrics, Liver and Solid Organ Transplantation Unit IRCCS ISMETT Palermo Italy; ^3^ UPMC Italy Palermo Italy; ^4^ Pediatria PO Agrigento Agrigento Italy; ^5^ Pronto Soccorso PO Agrigento Agrigento Italy

Maritime migration poses severe health risks, including respiratory intoxications caused by the inhalation of fuel vapours mixed with saltwater aerosols [1]. However, in late 2025, referral centres in Sicily identified a novel clinical cluster presenting with systemic toxicity distinct from classic hydrocarbon intoxication [2]. We reported a series of 16 intoxicated patients (Table 1, age 5–44 years), mostly characterised by predominant conjugated hyperbilirubinaemia with cholestatic pattern, in some cases associated with methaemoglobinaemia (MetHb).

**TABLE 1:** Clinical and laboratory characteristics of 16 patients presenting with acute toxic exposure during Mediterranean Sea migration (October–November 2025).

Patient ID	Age (years)	Sex	Total bilirubin (mg/dL)	Direct bilirubin (mg/dL)	MetHb (%)	AST (U/L)	ALT (U/L)	GGT (U/L)	INR	Symptoms	Outcome
P1	16	M	3.26	1.13	Positive at arrival	76	Normal	Normal	< 1.5	Acute kidney injury; buttock burns (fuel)	Recovered
P2	27	M	1.83	0.87	—	Normal	Normal	Normal	< 1.5	Haemoperitoneum (splenic rupture)	Recovered (splenectomy)
P3	25	M	2.80	1.38	59.2%	91	Normal	Normal	< 1.5	Haemolytic anaemia	Recovered
P4	21	M	—	—	11.4%	—	—	—	< 1.5	Anaemia	Recovered
P5	—	M	—	—	Positive	—	—	—	< 1.5	Shock; anaemia; fever	Recovered
P6	16	M	7.66	4.63	Positive	409	299	284	< 1.5	Anaemia; jaundice; fever	Recovered
P7	18	M	2.74	1.21	71.1%	Normal	Normal	Normal	< 1.5	Haemolytic anaemia; splenic rupture	Recovered (splenectomy)
P8	29	M	5.43	3.00	0.56%	192	Normal	Normal	< 1.5	Acute kidney injury; haemolysis; splenic rupture	Recovered (splenectomy)
P9	15	M	3.00	2.26	64.9%	216	107	102	2.62	Acute kidney injury; haemolysis; thrombocytopenia; fever	Recovered
P10	44	M	—	—	5.8%	—	—	—	< 1.5	Acute kidney injury	Recovered
P11	18	M	4.80	0.53	—	Normal	Normal	Normal	< 1.5	Haemolytic anaemia; splenic rupture	Recovered (splenectomy)
P12	25	M	—	—	41.3%	—	—	—	< 1.5	Haemolytic anaemia	Recovered
P13	34	M	9.10	7.74	Normal	57	129	483	< 1.5	Acute kidney injury; haemolytic anaemia; fever; jaundice	Recovered
P14	7	M	3.00	2.23	Normal	183	460	566	< 1.5	Asymptomatic	Recovered
P15	5	M	2.10	1.08	Normal	220	396	295	< 1.5	Asymptomatic	Recovered
P16	14	M	14.30	12.80	Positive at arrival	219	260	259	< 1.5	Fever; jaundice; abdominal pain	Recovered

*Note:* Data were collected during emergency humanitarian response; missing values (indicated by ‘—’) reflect the constraints of acute care settings. ‘Normal’ entries indicate values within institutional reference ranges; ‘Positive’ for MetHb indicates qualitative screening positive result without numerical quantification.

Abbreviations: ALT, alanine aminotransferase; AST, aspartate aminotransferase; GGT, gamma‐glutamyl transferase; INR, international normalised ratio; MetHb, methaemoglobin.

Between October and November 2025, several patients from multiple independent vessels departing from Libya presented following inhalation exposure in confined boat holds. Unlike standard gasoline intoxication, which primarily affects the lungs and central nervous system, these patients exhibited a ‘yellow’ phenotype, with unexpected severe jaundice. Laboratory analysis revealed a dissociation between bilirubin levels (severe elevation, median 3.13 mg/dL, range 1.83–14.30 mg/d; data available for 12 out of 16 patients) and aminotransferases (mild to moderate elevation). Conjugated hyperbilirubinaemia predominated in most cases, with a direct‐to‐total bilirubin mean ratio of 0.57. Fourteen of 16 patients were tested for MetHb, of whom ten showed elevated or positive results: six showed quantitative elevations ranging from 5.8% to 71.1%, while four had qualitative positive screening tests. Patients with elevated MetHb received methylene blue treatment upon arrival. While inhalation of aromatic hydrocarbons (e.g., gasoline fumes) can occasionally produce systemic toxicity, including hepatic injury, they rarely induce acute severe cholestasis [3]. This constellation suggests possible exposure to amino‐ or nitro‐aromatic compounds (e.g., aniline, nitrobenzene), known to cause hepatotoxicity and potentially used as illicit fuel octane boosters [4].

We highlight the case of a 14‐year‐old male (P16, Table 1) presenting with severe jaundice (total bilirubin 14.30 mg/dL, direct bilirubin 12.80 mg/dL, 89.5% conjugated), who tested for MetHb upon arrival and received methylene blue therapy. Viral and autoimmune screens were negative. Percutaneous liver biopsy revealed severe hepatocanalicular cholestasis with inspissated bile thrombi in dilated canaliculi. Hepatocytes showed diffuse feathery degeneration, a morphological hallmark of detergent‐like injury caused by retained bile salts. The biopsy also demonstrated a brisk ductular reaction with mild fibrosis (Ishak score 1). This cholestatic pattern may confirm a direct, acute toxic injury to the biliary transporters, distinct from infectious or autoimmune hepatitis.

Two young siblings from Sudan (ages 5 and 7 years, patients P15 and P14, respectively), who arrived in Lampedusa on November 8th, 2025, shared the same exposure environment with their parents on board. According to clinical reports from the initial receiving facility, both parents developed critical illness (mother: fulminant hepatic and renal failure requiring dialysis; father: severe cholestasis with acute kidney injury), while the two children remained largely asymptomatic despite persistent mild biochemical cholestasis (total bilirubin 2.10 and 3.00 mg/dL, respectively, with direct‐to‐total bilirubin ratios of 0.51 and 0.74). This family cluster, considering a comparable exposure, may suggest an age‐dependent toxicity gradient. We acknowledge that the maternal case (not included) was not transferred to our centres, representing a limitation of this preliminary report.

This report has important limitations: (1) Data collection occurred during an emergency humanitarian response without standardised research protocols; (2) key cholestatic markers (ALP) were not systematically measured; (3) methaemoglobin quantification was inconsistent, with some patients having only qualitative screening; (4) G6PD status was not assessed despite clinical relevance in populations from endemic regions and potential risk with methylene blue therapy; (5) temporal relationship between exposure and laboratory sampling was not systematically recorded; (6) fuel composition analysis was not feasible. These constraints reflect the reality of acute humanitarian medical care in overwhelming circumstances, and this report should be considered an early safety alert within an emergency context.

Despite these limitations, we believe this preliminary alert is justified: the clinical phenotype (predominant conjugated hyperbilirubinaemia with methaemoglobinaemia) is highly unusual for standard hydrocarbon exposure and suggests a novel toxic adulterant in fuels used for Mediterranean migration routes. Younger patients may present with milder clinical manifestations due to metabolic immaturity, while adolescents and adults appear to be at risk of severe injury mimicking biliary obstruction. An urgent, coordinated analysis of fuel composition along migration routes is required to confirm the presence of previously unrecognised adulterating agents and inform future emergency response protocols.

These cases may point to a previously unrecognised toxic syndrome affecting recent migrants during Mediterranean Sea crossings. While young children may appear clinically mild due to metabolic immaturity, adolescents and adults are at risk of severe injury mimicking biliary obstruction. This report serves as an emerging safety alert rather than a definitive epidemiological study, given the limitations inherent to emergency humanitarian care, including incomplete data capture, lack of standardised time points and constraints on systematic toxicological investigation. An urgent and updated analysis of fuel composition on migration routes is required to confirm the presence of new adulterating agents.

## Author Contributions

**Antonio Corsello:** conceptualization, methodology, data curation, investigation, resources, writing – original draft, writing – review and editing. **Simone Maria Calogero Gramaglia:** data curation, investigation, visualization. **Giuseppe Gramaglia:** data curation, validation, visualization. **Gerlando Verruso:** data curation, investigation. **Giusy Ranucci:** conceptualization, methodology, data curation, investigation, validation, writing – review and editing.

## Funding

The authors have nothing to report.

## Conflicts of Interest

The authors declare no conflicts of interest.

## Data Availability

The data that support the findings of this study are available from the corresponding author upon reasonable request.
